# Regulation of Chemokine Production via Oxidative Pathway in HeLa Cells

**DOI:** 10.1155/2009/183760

**Published:** 2010-01-27

**Authors:** Shinichiro Kina, Toshiyuki Nakasone, Hiroyuki Takemoto, Akira Matayoshi, Shoko Makishi, Nao Sunagawa, Feixin Liang, Thongsavanh Phonaphonh, Hajime Sunakawa

**Affiliations:** Department of Clinical Neuroscience Oral and Maxillofacial Functional Rehabilitation, University of Ryukyus, 207 Uehara, Nishihara, Okinawa 903-0215, Japan

## Abstract

Inflammation is associated with disease progression and, by largely unknown mechanisms, has been said to drive oncogenesis. At inflamed sites, neutrophils deploy a potent antimicrobial arsenal that includes proteinases, antimicrobial peptides, and ROS. Reactive oxygen species (ROSs) induce chemokines. In the present study, the concentrations of IL-8 in culture supernatants of HeLa cells treated with ROS were determined by enzyme-linked immunosorbent assay. We used *o*-phenanthroline to deplete Fe^2+^ in order to investigate the mechanisms through which ROSs induce IL-8 secretion in our system. The iron chelator *o*-phenanthroline effectively inhibited H_2_O_2_-induced ERK2 activation. Enzyme-linked immunosorbent assays showed that IL-8 protein secretion was elevated in ROS-treated HeLa cells. When Fe^2+^ was removed from these cells, IL-8 secretion was inhibited. Collectively, these results indicate that Fe^2+^-mediated Erk pathway activation is an important signal transduction pathway in ROS-induced IL-8 secretion in epithelial cells.

## 1. Introduction

Inflammation is associated with disease progression and, by largely unknown mechanisms, has been said to lead to drive oncogenesis. At inflamed sites, neutrophils deploy a potent antimicrobial arsenal that includes proteinases, antimicrobial peptides, and ROS [1]. Although ROSs are potently antimicrobial by virtue of their ability to kill microbial pathogens, in chronic inflammation, the continued production of ROS by neutrophils causes extensive tissue damage. Traditionally, this has been considered as random damage to cellular components [2]. Recently, ROSs have emerged as signal transduction molecules [3, 4]. 

In inflammatory cells, ROSs contribute to the expression of a variety of different inflammatory cytokines, adhesion molecules, and enzymes by activating redox-sensitive transcription factors such as nuclear factor-kB (NF-kB) or the AP-1 pathway [3, 5, 6]. In human monocytes, IL-8 production is induced by ROS, including by H_2_O_2_ via Erk-activated NF-kB [6].

Moreover, metal ion, such as iron, may take part in an important role in IL-8 production. The levels of iron in the cell seem to be delicately balanced, as iron loadings lead to free radical damage by the Fenton reaction [3]. In this study, our experiments were designed to determine the role of metal ions in the production of IL-8 by epithelial cells treated with ROS.

IL-8 has a molecular weight of 8.5 kDa and is of clinical significance in oral cancer diagnosis [7]. Oral cancer, the sixth most common cancer in the world, comprises the largest number of cancers in the head and neck category. The survival rate of oral cancer patients is 60%–80% when detected during its early stages; however, this number drops to 30%–40% when the cancer is diagnosed during advanced stages [8]. Identifying molecular markers of early disease can aid in its early diagnosis, which can improve the prognosis [9]. IL-8, as a salivary biomarker for early-stage oral squamous cell carcinoma (OSCC), was discovered through tissue-based expression profiling [7, 10]. Moreover, IL-8 plays a pivotal role in tumor angiogenesis [11, 12]. Fujimoto et al. reported a significant correlation between microvessel counts and interleukin- (IL-)8 levels in uterine cervical cancer [13]. 

Herein, we show that iron controls H_2_O_2_-induced chemokine expression in epithelial cells. In HeLa cells, hydrogen peroxide (H_2_O_2_) causes iron oxidation and amplifies Erk signaling. This elicits the expression of the chemokine interleukin-8 (IL-8).

## 2. Methods

### 2.1. Reagents

EGTA, PD98059 (p44/42 MAPKK inhibitor) and *o*-phenanthroline were purchased from Sigma-Aldrich (St Louis, MO, USA).

### 2.2. Cell Culture

The human cervical cancer cell line HeLa was routinely cultured in RPMI 1640 (Gibco) medium supplemented with 5% fetal bovine serum (Sigma), 40 units/mL penicillin, and 40 mg/mL streptomycin at 37°C under 5% CO_2_. Serum starvation was achieved by incubation in RPMI medium containing 0.5% fetal bovine serum for at least 16 hours prior to the direct addition of H_2_O_2_ to this culture medium. To observe the effect of Erk pathway inhibitors and agents to chelate Fe^2+^ on the secretion of IL-8 by HeLa cells, *o*-phenanthroline (0.2 mM), and mannitol (100 mM) were added to the culture medium 45 minutes before the direct addition of H_2_O_2_. Following further culturing for 12 hours, the supernatants from HeLa cells were collected and analyzed.

### 2.3. RT-PCR

Total RNA was extracted using Trizol (Invitrogen). The reverse-transcription of RNA to cDNA was performed using the RNA LA PCR Kit (TaKaRa). Expression levels of IL-8 and GAPDH mRNA in the HeLa cells were determined by RT-PCR using specific primers (invitrogen): 5′-CTGATTTCTGCAGCTCTGTG-3′ (sense) and 5′-TTCACTGGCATCTTCACTG-3′ (anti-sense) for IL-8, and 5′-CAGGGCTGCTTTTAACTCTG-3′ (sense) and 5′-GATGATCTTGAGGCTGTTGTC-3′ (anti-sense) for GAPDH. Temperature cycles were as follows: 94°C for 1 minute, followed by 28 cycles at 94°C for 30 seconds, 60°C for 30 seconds, and 72°C for 35 seconds for CXCL8, and 20 cycles at 94°C for 30 seconds, 55°C for 30 seconds, and 72°C for 35 seconds for GAPDH. The primers used for real-time PCR are 5′-ACTCCAAACCTTTCCACCC-3′ (sense) and 5-AAACTTCTCCACAACCCTCTG-3′ (antisense) for IL-8, and 5-GAAGGTGAAGGTCGGAGTC-3′ (sense) and 5′-GAAGATGGTGATGGGATTTC-3′ (antisense) for human GAPDH mRNA. SYBR Green PCR master mixes on the 7000 real-time PCR system (Applied Biosystem).

### 2.4. Western Blot Analysis

We determined Erk activation, by Western blotting with mouse monoclonal phospho-Erk1/2-specific antibody (Cell Signaling) using the ECL system (Amersham Pharmacia Biotech). Cells were washed once with PBS and lysed in TNE lysis buffer (10 mM Tris-HCl (pH7.8), 150 mM NaCl, 1 mM EDTA, and 1% NP40 ) supplemented with a protease and phosphatase inhibitor cocktail (Roche) for 10 minutes on ice. After centrifugation at 15,000 rpm for 10 minutes, lysates were subjected to 10% SDS-PAGE and electrotransferred onto a nitrocellulose membrane. The total amount of Erk2 was detected by using mouse monoclonal Erk2-specific antibody (Santa Cruz Biotech).

### 2.5. Determination of IL-8 Concentration

We determined the concentrations of IL-8 from HeLa cells by ELISA according to the manufacturer's instructions (Biolegend).

### 2.6. Statistical Analyses

All data are expressed as means ± s.e.m. We accumulated data for each condition from at least three independent experiments. We evaluated the significance using Student's *t*-test for comparisons between two mean values. We carried out multiple comparisons between more than three groups with ANOVA followed by the Tukey-Kramer test.

## 3. Results

### 3.1. H_2_O_2_ Activates ERK and Induces IL-8 Expression

**Figure 1:**
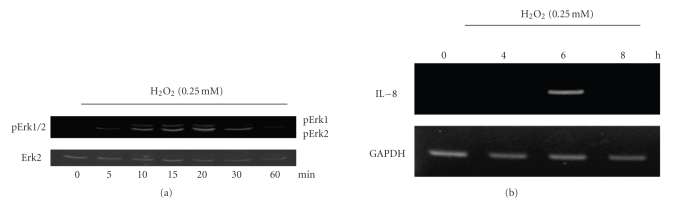
Time course of the activation of Erk and IL-8 production by H_2_O_2_. (a) Activated Erk: phosphorylated Erk1/2 induced by 0.25 mM H_2_O_2_ at the indicated time points of treatment. (b) Expression levels of IL-8 mRNA induced by incubation with 0.25 mM at the indicated time points.

Guyton et al. previously demonstrated that H_2_O_2_ activated Erk2 based on the direct measurement of kinase activity by employing the immune complex kinase assay [4]. However, based on other reports showing that Erk1 is more strongly activated than Erk2 after treatment with H_2_O_2_ [6], we would expect Erk1 to be a more activated kinase in our system in the presence of same stimulation, as described in other reports. To observe Erk activity, we assessed the level of phospho-Erk after H_2_O_2_ treatment. As shown in Figure 1(a), H_2_O_2_ stimulated the rapid and transient phosphorylation of Erk in HeLa cells. Erk activation was induced by H_2_O_2_ within 5 minutes for Erk2 and 10 minutes for Erk1, reaching maximum levels after 10–20 minutes (Figure 1(a)). A rapid inactivation of ERK2 then took place, with a return to basal Erk2 levels occurring within 60 minutes of H_2_O_2_ exposure. Unexpectedly, Erk2 becomes more phosphorylated than Erk1 in HeLa cells after H_2_O_2_ treatment.

In epithelial cells, ROSs contribute to the expression of a variety of different inflammatory cytokines by activating redox-sensitive transcription factors such as AP-1 [5]. We investigated the effect of H_2_O_2_ on the expression of IL-8. As shown in Figure 1(b), H_2_O_2_ treatment stimulated a transient but marked increase in the mRNA expression of IL-8.

### 3.2. Effect of Erk Activation on IL-8 Production following H_2_O_2_ Exposure

**Figure 2:**
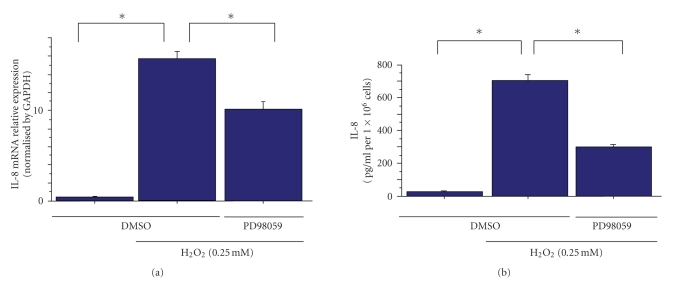
Erk controls H_2_O_2_-induced IL-8 expression in HeLa cells. The inhibitory effect of PD98059 on H_2_O_2_-induced expression (0.25 mM) of IL-8 mRNA ((a) real-time PCR) and protein secretion ((b) ELISA). Data points are means ± s.e.m. *P<.01 (*n* = 3).

In HeLa cells, treatment with H_2_O_2_ induced the mRNA expression of IL-8 (Figure 1(b)). In further experiments, we used ELISA to measure the IL-8 concentrations in culture supernatants from HeLa cells treated with H_2_O_2_. Consistent with previous reports, IL8 mRNA expression and secretion were suppressed by the Erk pathway inhibitor PD98059 (Figure 2) [14–16]. These results show that the Erk pathway controls H_2_O_2_-induced IL-8 production in HeLa cells.

### 3.3. Role of Free Radicals in Initiation of Erk Signaling and IL-8 Production Caused by H_2_O_2_

**Figure 3:**
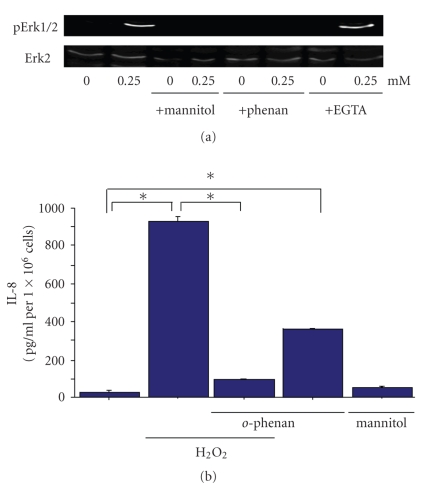
The role of free radicals in H_2_O_2_-mediated Erk2 activation and IL-8 production. (a) Mannitol (100 mM), *o*-phenanthroline (0.2 mM), or EGTA (2.5 mM) was added 45 minutes before direct addition of H_2_O_2_, and cells were harvested 15 minutes later for the analysis of p-Erk1/2 activity for the western blot. The inhibitors alone did not activate Erk1/2. (b) The inhibitory effects of *o*-phenanthroline on H_2_O_2_-induced expression (0.25 mM) of IL-8 protein secretion (ELISA) *P<.01 (*n* = 3).

The chemical signal generated by H_2_O_2_ that initiates the ERK cascade was also investigated. The iron chelator *o*-phenanthroline effectively inhibited ERK activation caused by H_2_O_2_ (Figure 3(a)), suggesting that metal-dependent reactions are required for kinase activation by H_2_O_2_. In the presence of metal ions, H_2_O_2_ can undergo conversion via dismutation reactions to other oxygen-derived free radical species, including the hydroxyl radical [17]. Indeed, mannitol, a free radical scavenger with specificity for hydroxyl radicals [4], also blocked H_2_O_2_-mediated ERK2 activation. Taken together, these results suggest that H_2_O_2_ undergoes metal-catalyzed conversion to a hydroxyl radical-like species, and that oxidation by this free radical initiates signal transduction leading to ERK activation by H_2_O_2_. In contrast, H_2_O_2_-induced ERK activation was unaffected by the calcium chelator, EGTA.

Fe^2+^ ion has been implicated in the activation of the Erk pathway. Therefore, it was considered possible that Fe^2+^ ion transduces the regulation of H_2_O_2_-induced IL-8 secretion. To address this, we investigated whether the depletion of Fe^2+^ reduced IL-8 secretion from HeLa cells treated with H_2_O_2_. HeLa cells were treated with 200 *μ*M of *o*-phenanthroline or cultured in serum starved medium for 45 minutes prior to H_2_O_2_ exposure. H_2_O_2_-induced IL-8 production in HeLa cells was inhibited by the removal of Fe^2+^ (Figure 3(b)), suggesting that Fe^2+^ ion is key in H_2_O_2_-induced IL-8 production.

 Taken together, these results indicate that the levels of IL-8 produced by HeLa cells are predominantly controlled by the oxidative pathway.

Surprisingly, *o*-phenanthroline itself induced IL-8 secretion. So, we concluded that iron balance is very important for IL-8 production.

## 4. Discussion

It has been postulated that chemokine production by monocytes in response to H_2_O_2_ requires Erk pathway activation via Ca^2+^ channel-mediated Ca^2+^ influx [6, 18]. However, our studies on epithelial cells seemed to indicate no inhibitory effect on Erk pathway activation after the removal of extracellular Ca^2+^ on treating HeLa cells with H_2_O_2_. In this study, we examined the inhibitory effects of other metal ion chelators on IL-8 production in epithelial cells in response to H_2_O_2_, with the aim of elucidating the nature of the hydroxyl radical reaction. Thus, by varying the metal ion chelator, we can obtain new insights into role of other metal ions in chemokine production. Furthermore, these studies have important implications regarding optimal parameters for oral cancer screening [19].

Under conditions in which H_2_O_2_ is maintained at a relatively low level (250 *μ*M), it did not induce cell death, as described in other reports [6, 20]. The primary determinant of chemokine production is Erk activation, and, hence, HeLa cells express mRNA of IL-8 on H_2_O_2_ treatment. However, it is likely that the nature of Erk pathway activation, such as Erk1 or Erk2 specificity, slightly differs between monocytes and HeLa cells. 

Under conditions in which monocytes are exposed to H_2_O_2_, Ca^2+^ influx occurs through Ca^2+^ channels, and, as a result, the Erk pathway, especially Erk1, is activated [6]. After the removal of Ca^2+^ using EGTA, Erk1 activation with H_2_O_2_treatment is inhibited in monocytes [6]. On the contrary, when HeLa cells are exposed to H_2_O_2_ at the same level (250 *μ*M), the Erk pathway, especially Erk2, is activated.

We predicted that the suppression of IL-8 transcription inhibits the subsequent steps in IL-8 secretion. To test this, we pretreated H_2_O_2_-exposed HeLa cells with PD98059 and measured IL-8 secretion. Although the H_2_O_2_-exposed HeLa cells showed an increase in IL-8 secretion, this secretion was significantly inhibited in PD98059-treated HeLa cells. These results support the proposal that Erk mediates IL-8 secretion.

 In an effort to define the mechanism by which H_2_O_2_ regulates chemokine production, we examined the inhibitory effect of metal ion chelators on Erk pathway activation. Whereas EGTA showed no inhibitory effect on Erk pathway activation in HeLa cells, the level of phospho-Erk in HeLa cells pretreated with *o*-phenanthroline was markedly lower.

 The crucial difference in experimental conditions between U937 monocytes and HeLa epithelial cells is the cellular character. In terms of the molecular expression pattern, such as receptors, these cells are very different. Epithelial cells express EGFR, which is capable of being activated by H_2_O_2_ [21, 22]. Conversely, monocytes do not express EGFR. Consequently, the H_2_O_2_ receptor may be different between monocytes and epithelial cells. 

 We subsequently examined the inhibitory effect of *o*-phenanthroline on IL-8 production in HeLa cells. IL-8 production was upregulated in H_2_O_2_-treated HeLa cells. In contrast, its production was significantly downregulated in H_2_O_2_-treated HeLa cells with in comparison to those without* o*-phenanthroline. These results demonstrate that the *o*-phenanthroline-dependent downregulation of Erk pathway is sufficient to suppress IL-8 production in epithelial cells upon H_2_O_2_-mediated inflammation.

The iron balance is another key determinant in chemokine production. As Fe^2+^, iron is a stronger redox molecule than calcium, an effect that is likely mediated by an increase in the presence of iron. However, the shortage of iron affects the observed IL-8 production in very different ways. In the case of *o*-phenanthroline or DFO itself, a chelator for Fe ion, induced IL-8 production in epithelial cells [23]. The mechanism by which DFO or *o*-phenanthroline induces IL-8 production has remained unknown. 

Inflammation is associated with disease progression. Recent reports have described an H_2_O_2_-independent role of metal ions in chemokine production in epithelial cells [5]. However, the role of the oxidative pathway in chemokine production has until now remained unknown. Herein, we have shown that H_2_O_2_-induced chemokine production in epithelial cells is increased owing in part to the H_2_O_2_-mediated oxidation of iron.

## References

[1] Fialkow L, Wang Y, Downey GP (2007). Reactive oxygen and nitrogen species as signaling molecules regulating neutrophil function. *Free Radical Biology and Medicine*.

[2] Henricks PAJ, Nijkamp FP (2001). Reactive oxygen species as mediators in asthma. *Pulmonary Pharmacology and Therapeutics*.

[3] Dröge W (2002). Free radicals in the physiological control of cell function. *Physiological Reviews*.

[4] Guyton KZ, Liu Y, Gorospe M, Xu Q, Holbrook NJ (1996). Activation of mitogen-activated protein kinase by H_2_O_2_: role in cell survival following oxidant injury. *Journal of Biological Chemistry*.

[5] Kim Y-M, Reed W, Wu W, Bromberg PA, Graves LM, Samet JM (2006). Zn^2+^-induced IL-8 expression involves AP-1, JNK, and ERK activities in human airway epithelial cells. *American Journal of Physiology*.

[6] Yamamoto S, Shimizu S, Kiyonaka S (2008). TRPM2-mediated Ca^2+^ influx induces chemokine production in monocytes that aggravates inflammatory neutrophil infiltration. *Nature Medicine*.

[7] St. John MAR, Li Y, Zhou X (2004). Interleukin 6 and interleukin 8 as potential biomarkers for oral cavity and oropharyngeal squamous cell carcinoma. *Archives of Otolaryngology—Head and Neck Surgery*.

[8] Franzmann EJ, Reategui EP, Carraway KL, Hamilton KL, Weed DT, Goodwin WJ (2005). Salivary soluble CD44: a potential molecular marker for head and neck cancer. *Cancer Epidemiology Biomarkers & Prevention*.

[9] Sidransky D (2002). Emerging molecular markers of cancer. *Nature Reviews Cancer*.

[10] Tan W, Sabet L, Li Y (2008). Optical protein sensor for detecting cancer markers in saliva. *Biosensors and Bioelectronics*.

[11] Arenberg DA, Kunkel SL, Polverini PJ, Glass M, Burdick MD, Strieter RM (1996). Inhibition of interleukin-8 reduces tumorigenesis of human non-small cell lung cancer in SCID mice. *Journal of Clinical Investigation*.

[12] Mizukami Y, Jo W-S, Duerr E-M (2005). Induction of interleukin-8 preserves the angiogenic response in HIF-1*α*-deficient colon cancer cells. *Nature Medicine*.

[13] Fujimoto J, Sakaguchi H, Aoki I, Tamaya T (2000). Clinical implications of expression of interleukin 8 related to angiogenesis in uterine cervical cancers. *Cancer Research*.

[14] Josse C, Boelaert JR, Best-Belpomme M, Piette J (2001). Importance of post-transcriptional regulation of chemokine genes by oxidative stress. *Biochemical Journal*.

[15] Zeng X, Dai J, Remick DG, Wang X (2003). Homocysteine mediated expression and secretion of monocyte chemoattractant protein-1 and interleukin-8 in human monocytes. *Circulation Research*.

[16] Chiu L-L, Perng D-W, Yu C-H, Su S-N, Chow L-P (2007). Mold allergen, Pen C 13, induces IL-8 expression in human airway epithelial cells by activating protease-activated receptor 1 and 2. *Journal of Immunology*.

[17] Cadenas E (1989). Biochemistry of oxygen toxicity. *Annual Review of Biochemistry*.

[18] Méndez-Samperio P, Palma-Barrios J, Vázquez-Hernández A, García-Martínez E (2004). Secretion of interleukin-8 by human-derived cell lines infected with *Mycobacterium bovis*. *Mediators of Inflammation*.

[19] Katakura A, Kamiyama I, Takano N (2007). Comparison of salivary cytokine levels in oral cancer patients and healthy subjects. *The Bulletin of Tokyo Dental College*.

[20] Hara Y, Wakamori M, Ishii M (2002). LTRPC2 Ca^2+^-permeable channel activated by changes in redox status confers susceptibility to cell death. *Molecular Cell*.

[21] Zhougang S, Schnellmann RG (2004). H_2_O_2_-induced transactivation of EGF receptor requires Src and mediates ERK1/2, but not Akt, activation in renal cells. *American Journal of Physiology*.

[22] Bae YS, Kang SW, Seo MS (1997). Epidermal growth factor (EGF)-induced generation of hydrogen peroxide. Role in EGF receptor-mediated tyrosine phosphorylation. *Journal of Biological Chemistry*.

[23] Choi E-Y, Park Z-Y, Choi E-J (2007). Transcriptional regulation of IL-8 by iron chelator in human epithelial cells is independent from NF-*κ*B but involves ERK1/2- and p38 kinase-dependent activation of AP-1. *Journal of Cellular Biochemistry*.

